# Analyzing adaptive modulation in spinal motor neurons using multi-objective evolutionary algorithms

**DOI:** 10.1186/1471-2202-16-S1-P94

**Published:** 2015-12-18

**Authors:** Tomasz G Smolinski, Joseph Lombardo, Melissa A Harrington

**Affiliations:** 1 Department of Computer and Information Sciences, Delaware State University, Dover, DE 19901, USA; 2 Department of Biological Sciences, Delaware State University, Dover, DE 19901, USA

## 

Spinal motoneurons have long been thought to be simply part of a relay system that provides rapid, stereotyped outputs for muscles on the basis of a supraspinal plan tuned by sensory inputs, and activity-dependent plasticity (ADP) has been presumed to be a property of the brain. However, recent work indicates that ADP occurs in spinal motor neurons during development, as well as later in life with skills acquisition and maintenance, and in response to trauma and disease [1]. Understanding how spinal motoneuron output can be modified by both increased and decreased activity is thus a fundamental challenge with implications for athletic training, rehabilitation, and advanced prosthetics. We hypothesize that that alteration in the function of Kv7.2 channel (which carries the M current) and changes in axonal initial segment (AIS) properties are the primary mechanisms of adaptation of spinal motoneurons to prolonged network activation. This hypothesis is supported by the literature and our experimental data demonstrating that persistent activation of spinal cord networks decreases spinal motoneuron output in a manner consistent with the enhancement of a sub-threshold, non-inactivating potassium conductance. KCNQ/Kv7 channels, which are non-inactivating potassium channels that activate in the sub-threshold range [2], are expressed at the AIS, nodes of Ranvier, and soma of spinal motoneurons [3,4], and modulate their excitability [5,6].

To test our hypothesis, we developed a realistic computational model of spinal motoneuron activity before and after persistent network activation. As the starting point, we utilized a reconstructed spinal motoneuron morphology of neonatal mice [7] together with the detailed specification of the active and passive somatodendritic and axonal properties derived from a rodent cortical neuron model [8]. The model parameter values were adjusted to match our recordings of motoneuron electrophysiological properties using a multi-objective evolutionary algorithm (MOEA) [9]. The algorithm matches multiple selection criteria simultaneously (*e.g*., spike frequency, shape, adaptation rate, *etc*.) and generates entire collections of neuronal models that can be mined for rules describing the phenomena captured by the models (for instance, co-regulations between ionic conductances). Furthermore, since the MOEA generates two independent databases of models (*i.e*., before and after persistent activation), we are able to directly compare the phenomena discovered by our data mining process in each dataset, thus elucidating the mechanisms underlying plasticity.
